# Utilization of nano-olive stones in environmental remediation of methylene blue from water

**DOI:** 10.1007/s40201-019-00438-y

**Published:** 2020-01-21

**Authors:** Mohammad A. Al-Ghouti, Samah S. Dib

**Affiliations:** Department of Biological and Environmental Sciences, College of Arts and Sciences, Qatar University, P.O. Box: 2713, Doha, Qatar

**Keywords:** Olive stones, Nano-olive stones, Adsorption, Methylene blue, Water treatment

## Abstract

**Background:**

The use of agricultural waste as a low-cost adsorbent for the removal of hazardous methylene blue (MB) from aqueous solution was investigated. In this research, the potentiality of using black nano olive stones (black NOS) and green nano olive stones (green NOS) for MB adsorption was conducted.

**Methods:**

Various remediation parameters such as initial MB concentration, pH, and temperature were investigated. Thermodynamic study was carried out to determine the homogeneity of the adsorbent and spontaneity of the adsorption process. Different physical and chemical characterizations were studied using scanning electron microscopy (SEM), Fourier transform infrared (FTIR), Brunauer-Emmett-Teller (BET) surface area, pore radius and pore volume.

**Results:**

It was found that NOS exhibits an acidic nature, however the highest MB removal efficiency was recorded at pH 10; reaching up to 71%. The negative value of the heat of the adsorption process (*∆H* ° ) indicated the reaction followed an exothermic pathway while the negative value of Gibbs adsorption (*∆G* ° ) further suggested its spontaneous nature. The results indicated that the Freundlich model described well the adsorption process with 99.5% correlation coefficient for green NOS. FTIR was used to analyze functional groups on the adsorbents’ surfaces that could play vital roles in the remediation process. SEM analysis revealed that the adsorbents comprised of abundant spherical deep cavities and porous nature.

**Conclusion:**

The result obtained successfully demonstrated the potential of using black and green NOS as suitable adsorbents for the removal of MB from water.

## Introduction

The rapid growth in global population and industrialization have indeed put stress on the natural resources and put tremendous challenges to the ecosystem [1–3]. Since textile industry utilizes great amount of water, global water resources are facing a great stress. Consequently, controlling water pollution is necessary [1]. One of the growing concerns from textile industry includes high water consumption and discharge of polluted water to the environment. The main causes of pollution include bleaching, dying, and the use of different chemicals on the fabric in which around 10–15% of the used dyes passes into the effluents [2]. Usually, to process a ton of textile, around 150 m^3^ of water is utilized in which 80% of the industrial wastewater is discharged to the environment [3]. Typically, aqueous dyeing demands 100–180 L of water to dye 1Kg of fibers [4]. Textile industry is considered as one of the biggest polluters of clean water. Around 8000 different chemicals and more than 3000 various types of dyes are consumed by textile industry in various processes including bleaching, dyeing, printing, and finishing. Most of these chemicals either have a direct or indirect impact on human health, aquatic life or cause water or soil pollution. Furthermore, various studies have mentioned that 280,000 tons of textiles dyes are discharged as industrial effluent each year [5]. These effluents include heavy metals and toxic compounds, which pose direct threat to the aquatic life, plants, soil, and human health. However, textile dyes mostly affect the water bodies, leading to alteration of the watercolor, deterioration of the water quality, as well as harming the aquatic organisms [6].

Textile dyes affect the environment in many ways such as preventing the sunlight penetration, which might cause eutrophication. As a result, causing disturbance in water ecology due to the low photosynthesis rate [7]. Moreover, it is reported that some of the textile dyes are considered carcinogenic for human health and should be treated and remediated before being released into the water bodies [8]. The process involved in industrial dyeing are very complicated due to the presence of inorganic salts, surface-active agents, high alkali pH, fibers and dyes [9]. In addition, dyes contain chromophores and auxochromes, which are used to detect the type of the dye i.e. acidic, basic, disperse, reactive, azo, diazo and anthraquinone based on the chromophores [10]. Dyes are mainly characterized based on the ionic categorization, which strongly affects the efficiency of the adsorption. The non-ionic dyes are divided into vat and disperse dyes, while the ionic dyes categorized as cationic dyes, which are basic, and the anionic dyes, which are acidic reactive dyes. One of the dyes that is perhaps widely used in the textile industry is methylene blue (MB) dye (C_16_H_18_C_l_N_3_S), which is a cationic synthetic dye substance and does not occur naturally. It is present in the form of green/blue powder or crystalline solid, which dissociates in aqueous solutions as chloride ions and cations.

Additionally, many studies have been investigating the effectiveness of different materials for water remediation from MB including electrochemical treatment, coagulation/flocculation, aerobic and anaerobic treatments in order to meet the water quality standards. Dyes and their corresponding breakdown products are very difficult to remediate using traditional wastewater treatment systems [11]. In fact, many of the traditional methods were not successful in removing toxic pollutants from wastewater in low-energy requirement [12]. The used conventional treatment methods in textile dye removal can be classified as physical, chemical, and biological treatment methods. The difficulty in dye removal from aquatic media by conventional methods is due to the stability and the complex aromatic structure of the dyes. Therefore, adsorption process is considered as a simple method for the minimization of organic pollutants to the desired level where wastewater is drained into communal sewage or directly into water [13]. Wang et al. [14] stated that the adsorption process can achieve elimination of dye as high as 92.17%, and can reduce the chemical oxygen demand (COD) to 91.15% in series adsorption reactors.

Various agricultural waste materials can be used as an adsorbent for water and wastewater treatment including silica, activated carbon, metal oxides, clay materials, fruit seeds, and modified compounds in the form of composites [14–16]. Choosing suitable adsorbents depends on their availability, cost, toxicity, thermal stability and pore size. Olive stones (OS) are very famous and abundant agricultural by-product in the Mediterranean region and it is extensively cultivated fruit crop around the world [17]. OS is considered as biosorbent with high adsorption capacities towards many toxic compounds, good mechanical strength, and low ash content [18]. Furthermore, OS is lignocellulosic biomass, which has cellulose with varying content between 30 to 34%, hemicellulose content from 21 to 28%, and lignin from 21 to 25% as major components [19], in addition to significant amount of proteins and fats. As a consequence to its high carbon content, it can be converted to activated carbon by thermal decomposition, in which they are characterized for their high adsorptive properties [16, 20].

Several studies have highlighted the efficiency of OS to remove MB from the aquatic media [21, 22]. However, the efficiency of the process depends on various operating parameters, including pH, dye concentration, temperature, and particle size. Conventionally, adsorption on activated carbon (AC) derived from organic and inorganic materials has been excessively investigated to remediate different pollutants from water and wastewaters. Despite the popularity of AC in adsorption, it has poor regeneration capacities and its use and re-use is still costly. Accordingly, nanomaterials such as nano-AC are widely used in the field of environmental remediation due to their huge effects on remediation [23]. Nano-remediation has recently gained popularity, which deals with wastewater treatment applications, and it is considered very efficient, eco-friendly, and cost-effective due to the unique functionalities of the nanomaterials to treat industrial effluents [24]. During the remediation process, nanomaterials are used as adsorbent, which potentially remove pollutants from wastewater, and enhance the chemical and adsorption activities due to their small size and high surface area with better porosity characteristics [25–27]. Nanomaterials and commercial activated carbon have the same characteristics of large surface area, however, nanomaterials are synthesized easily at a lower cost, and they are required in a smaller amount for the effective pollutants’ removal. Hence, the objectives of this paper are: i) obtaining black and green OS in nanoparticle size to be used as an adsorbent; ii) characterize the adsorbents in terms of SEM, FTIR, BET, surface area, pore radius and pore volume; iii) to utilize the obtained nanoparticle adsorbents for adsorption of MB from aqueous medium and investigate their adsorption isotherms.

## Materials and methods

### Sample collection and preparation

Two representative samples of black OS and green OS were collected. The OS samples were initially washed with tap water, followed by distilled water several times to remove any impurities. After that, OS samples were dried under 150 °C for 24 h, and separately crushed into small pieces. Then, the obtained OS samples were ground into fine powder using CryoMill to obtain nanoparticles, namely black nano-olive stones (black NOS) and green nano-olive stones (green NOS). The powders were stored in containers in dry place.

### Characterization of adsorbents

The surface morphology of the prepared black NOS and green NOS were characterized and analyzed by scanning electron microscope and energy-dispersive X-ray spectroscopy (SEM-EDS-NOVA NANOSEM 450). The nano-size was detected and confirmed by using SEM magnification, which was between 100 and 200 nm. The chemical characterization of the functional group of the sample’s surface was performed by FTIR (FT-IR/FT-NIR Spectrometer- Spectrum 400). The carbon, hydrogen, and nitrogen (CHN) elemental analysis was carried out using FLASH 2000-Organic elemental analysis. The elemental composition of the samples was detected by carbon, hydrogen, and nitrogen (CHN) analyzer. Moreover, 0.03 g of each black NOS and green NOS were added to 30 mL of distilled water and then kept in the mechanical shaker for 24 h to record the solution pH using a digital pH meter (JENWAY Model 370 pH/mV Meter) before and after sample shaking. Furthermore, to determine the surface area and pore size distribution of the adsorbents, the samples were first dried in an oven at 100 °C for 1 h and then BET (model Aim Sizer-AM301) was used for the analysis. Bulk density of the adsorbent was determined by adding a known mass of the adsorbent gently to a cylinder without compaction. However, particle density was measured by adding a known mass into known volume of water into the graduated cylinder and the difference between water volume values was recorded. The mass and the change of volume were recorded, and the two densities (g/mL) and porosity (%) were calculated using the following equations:

1 $$ \mathrm{Density}=\mathrm{mass}\ \left(\mathrm{g}\right)/\mathrm{change}\ \mathrm{in}\ \mathrm{volume}\ \left(\mathrm{mL}\right) $$

2 $$ \mathrm{Porosity}=1-\left(\mathrm{bulk}\ \mathrm{density}/\mathrm{particle}\ \mathrm{density}\right)\times 100\% $$

### Preparation of MB stock solution

Stock solution of MB was prepared by dissolving 1 g of MB in 1 L of distilled water. Various initial MB concentrations were prepared (50, 100, 200, 300, 400, 600, 800 and 1000 ppm). Blank was also included. MB calibration curve was conducted under 663 nm through measuring the absorbance for each concentration (Lambda 25 UV/VIS Spectrophotometer).

### Batch adsorption of methylene blue (MB)

Various remediation parameters were investigated including pH (2, 4, 6, 8, and 10), temperature (25, 35, and 45 °C) and initial concentration (50–1000 ppm). The pH was adjusted using 0.1 M of hydrochloric acid (HCl) or sodium hydroxide (NaOH). A 0.05 g of the adsorbent (green NOS and black NOS) and 50 mL of MB solution at different initial concentrations were placed in glass bottle and were shaken at 150 rpm by using a temperature-controlled shaker (Shaking Incubator, MODEL: SSI10R-2, Orbital-Shaking) for 24 h, then they were centrifuged at 40,000 rpm. All the samples were filtered, and MB concentration was determined using ultraviolet-visible (UV)-spectrophotometer (PerkinElmer Lambda 25 UV/VIS Spectrophotometer).

### Adsorption isotherm of methylene blue (MB)

Three models of adsorption isotherm were applied to understand the kinetics and mechanism of the adsorption process as shown in Table 1. The non-linear regression root means square error (RMSE), the sun of error squares (SSE), and Chi-squares (*x*^*2*^) were used as criterion for the quality of fitting; according to Eqs. 3–5, respectively.

**Table 1:** Adsorption isotherm models used in current study [27–29]

Model	Equation	Parameters
Langmuir adsorption isotherm	$\frac{C_e}{q_e}=\frac{1}{K_L.{Q}_o}+\frac{C_e}{Q_0}$	q_e_ is the amount of adsorbate in the adsorbent at equilibrium (mg/g). Q_0_ is the maximum monolayer coverage capacities (mg/g). K_L_ is the Langmuir isotherm constant (L/mg). C_e_ is the equilibrium concentration (mg/L).
Freundlich adsorption isotherm	$Log{q}_e= Log{K}_f+\frac{1}{n} Log{C}_e$	K_f_ is the Freundlich adsorption constant (mg/g)(L/g)^n^. The value of n indicates the type of isotherm. When $\frac{1}{\mathrm{n}}$ is greater than zero (0$<\frac{1}{\mathrm{n}}<1$), the adsorption is favorable, when $\frac{1}{\mathrm{n}}=$1, the adsorption is irreversible, and when $\frac{1}{\mathrm{n}}>1$ the adsorption is unfavorable.
Dubinin–Radushkevich adsorption isotherm	${q}_e={q}_D.\exp \left(-{B}_D{\left[R.T.\ln \left(1+\frac{1}{C_e}\right)\right]}^2\right)$ $\mathit{\ln}{q}_e=\mathit{\ln}{q}_D-{B}_D.{\varepsilon}_D^2$ $E=\frac{1}{\sqrt{2{B}_D}}$	*q*_*D*_ is the maximum adsorption capacity in (mg/g), *B*_*D*_ is the free energy coefficient of the adsorption (mol^2^/kJ^2^)

3 $$ RMSE=\sqrt{\frac{1}{N-2}x{\sum}_1^n{\left({q}_{e,\mathit{\exp}}-{q}_{e, cal}\right)}^2} $$

4 $$ SSE=\frac{1}{N}{\sum}_{n-1}^{\infty }{\left({q}_{e,\mathit{\exp}}-{q}_{e, cal}\right)}^2 $$

5 $$ {x}^2={\sum}_1^N\frac{\left({q}_{e,\mathit{\exp}}-{q}_{e, cal}\right)}{q_{e, cal}} $$

Where q_e,exp_ (mg/g) is the experimental adsorption capacity, q_e,cal_ is the calculated adsorption capacity using an isotherm model (mg/g), and N is the number of observations in the experiment.

### Statistical analysis

Single and two-way ANOVA were conducted to validate the experimental data of the effect of pH, initial MB concentration, and temperature on the adsorption of MB onto the adsorbents using Microsoft Excel for the results of adsorption experiment. Here, the effect of pH changes was studied using one-way ANOVA and the relation between the concentration and temperature was studied using two-way ANOVA. The coefficient of determination (R^2^) and Fisher’s statistical test (F test) were used to examine the quality and significant of regression equation. The R^2^ was used for judging the quality or goodness of the fitted quadratic models. The R^2^ and F statistic were determined by Eqs. 6 and 7 [28],

6 $$ {\displaystyle \begin{array}{l}{R}^2\frac{\sum \limits_{i=1}^n{\left({Y}_{pred,i}-{Y}_{obs, mean}\right)}^2}{\sum \limits_{i=1}^n{\left({Y}_{obs,i}-{Y}_{pred,i}\right)}^2+\sum \limits_{i=1}^n{\left({Y}_{pred,i}-{Y}_{obs, mean}\right)}^2}\\ {}\kern1em =\frac{\sum \limits_{i=1}^n{\left({Y}_{pred,i}-{Y}_{obs, mean}\right)}^2}{\sum \limits_{i=1}^n{\left({Y}_{pred,i}-{Y}_{obs, mean}\right)}^2}\end{array}} $$

7 $$ F=\frac{\sum \limits_{i=1}^n{n}_i{\left({Y}_{mean,i}-{Y}_{pred,i}\right)}^2/\left(n-p\right)}{\sum \limits_{i=1}^n\sum \limits_{j=1}^{n_i}{\left({Y}_{ij}-{Y}_{mean,i}\right)}^2/\left(N-n\right)} $$

Where *p* is the number of parameters in the model; *i* is an index of each of the *n* distinct *x* values; *j* is an index of the response variable observations for a given *x* value; *n*_*i*_ is the number of *Y* values associated with the *i*^*th*^*x* value; *Y*_*ij*_ is the experimental response of run *i*, replicate *j*; *Y*_*pred,i*_ is the response obtained from using the proposed polynomial at run *i* and *Y*_*mean,i*_ is the observed mean of all the replicates at run *i*.

## Results and discussion

### Chemical and physical characterization of the adsorbent

#### Scanning Electron Microscopy (SEM) analysis

**Fig. 1:**
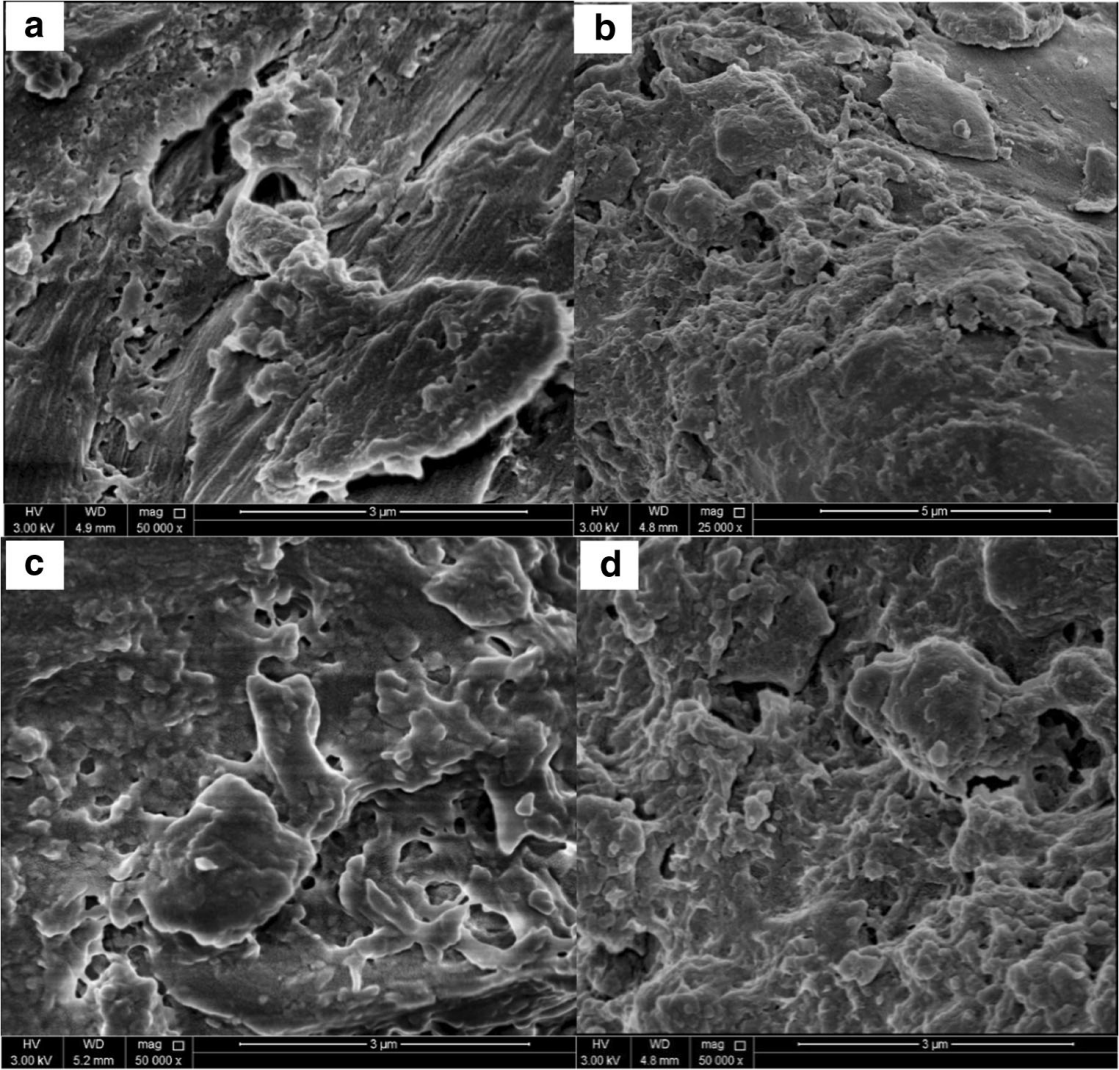
SEM images of (**a**) green NOS before MB adsorption, (**b**) green NOS after MB adsorption, (**c**) black NOS before adsorption, and (**d**) black NOS after adsorption of 600 ppm of MB dye at 25 °C

It can be seen form Fig. 1a, c that NOS consists of very fine spherical shape structure, as well as the presence of porous structure which created large surface area to interact with MB molecules and enhance the adsorption. The non-uniform surface and different sizes of both green and black NOS after adsorption process were shown in Fig. 1b, d, where the surface of the adsorbent is fully covered with MB dye. It has been shown that the surface of the adsorbent was fully changed, and the pore sizes reduced. A recent study that deals with walnut shell showed that the smaller particles of the shells have larger surface area than the bulk particles, resulting in more active sites, thus significantly improving the removal percentages of MB [8]. Tan et al. [30] showed that the adsorption capacity for cationic dye such as MB was almost as high as 59.7 mg/g and anionic acid red dye was 45.2 mg/g. Furthermore, it was also concluded that the surface structure and charge of the dyes played an important role in the adsorption capabilities of carbon nanotubes. Shakoor and Nasar [15], investigated adsorption of MB using *Citrus limetta* peel waste as low-cost adsorbent and found that as the particle size decrease, the removal efficiency increases from 93.5% to 98.2%.

#### CHN composition

OS is known to have lignocellulosic components, which are cellulose, hemicelluloses, holocelluloses, and lignin in varying amounts [31]. Table 2 shows the composition of both green and black NOS before and after adsorption of 400 ppm of MB at 25 °C, pH 10. Table 2 indicates the high carbon content for both green and black NOS as a major element, and very low nitrogen content. Green NOS has 52.59 %C, 6.47 %H, and 0.40 %N, while the black NOS sample has 48.76 %C, 6.18 %H, and 0.41 %N. The composition of OS agrees with the previously published data where the seed contains 45.54% of carbon, 6.28% of hydrogen, and 0.18% N [19]. It was also noted from Table 2 that the carbon content decreased from 52.59 to 45.80% for green NOS. Similarly, the black NOS showed a decrease in carbon percentage from 48.76 to 45.79% after MB adsorption; confirming the effectiveness of MB adsorption by the green NOS. Moreover, pore volume (cm^3^/g) of the samples were shown in Table 2.

**Table 2:** Elemental composition, pore size and surface area characterization of black and green NOS before and after adsorption using CHNS analyzer

Element	(% wt./wt., dry weight)			
	Green NOS		Black NOS	
	Before adsorption	After adsorption	Before adsorption	After adsorption
Carbon (C)	52.6 ± 0.5	45.8 ± 0.5	48.8 ± 0.5	45.8 ± 0.5
Hydrogen (H)	6.47 ± 0.11	6.27 ± 0.11	6.18 ± 0.11	6.28 ± 0.11
Nitrogen (N)	0.40 ± 0.05	0.80 ± 0.05	0.41 ± 0.05	0.84 ± 0.05
Pore size and surface area characterization				
	Volume of the macropores (cm^3^/g)	Volume of the mesopores (cm^3^/g)	Specific surface area (S_BET_ (m^2^/g)	
OS	0.171 ± 0.009	0.0355 ± 0.0011	1.03 ± 0.06	
Green NOS	0.111 ± 0.007	0.0564 ± 0.0014	10.55 ± 0.19	
Black NOS	0.099 ± 0.003	0.0646 ± 0.0019	11.89 ±0.38	

#### Fourier-transform infrared spectroscopy (FTIR) study

**Fig. 2:**
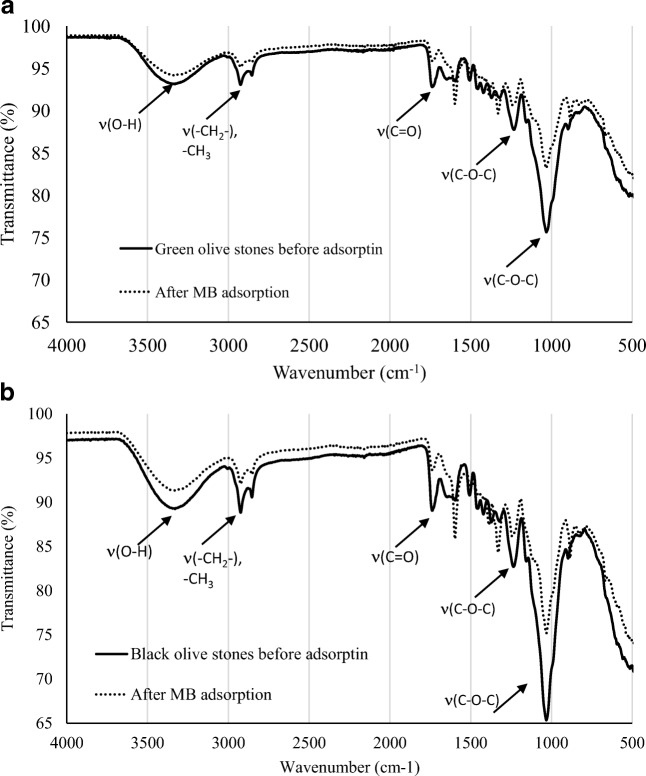
FTIR spectra of (**a**) green NOS before and after adsorption of MB 600 ppm, and (**b**) black NOS before and after adsorption of MB 600 ppm at pH = 10, 25 °C

Figure 2a shows the FTIR spectra of the green NOS before and after MB adsorption. The first broad peak stretching at 3330.92 cm^−1^ was identified as O-H hydroxyl with a single H bonded [32, 33]. The band at 2924.90 cm^−1^ indicates C-H alkane symmetric and asymmetric stretching. The two weak peaks that were detected at 3000 cm^−1^ were associated with the aromatic and aliphatic stretching of (C-H) mode [34]. Furthermore, C=O carbonyl stretching at 1738.61 cm^−1^ indicates the presence of hemicellulose [35]. In fingerprint region, strong band near 1231.21 cm^−1^ and 1030.97 cm^−1^ could be attributed to C-O stretching. After MB adsorption, the shift of bands were observed for O-H at 3339.71 cm^−1^, C-H stretching at 2923.12 cm^−1^, C=O stretching at 1740 cm^−1^, and in fingerprint region, the two strong bands also shifted to 1224.67 cm^−1^ and 1032.01 cm^−1^. This indicates the involvement of these functional groups in MB adsorption [36].

**Fig. 3:**
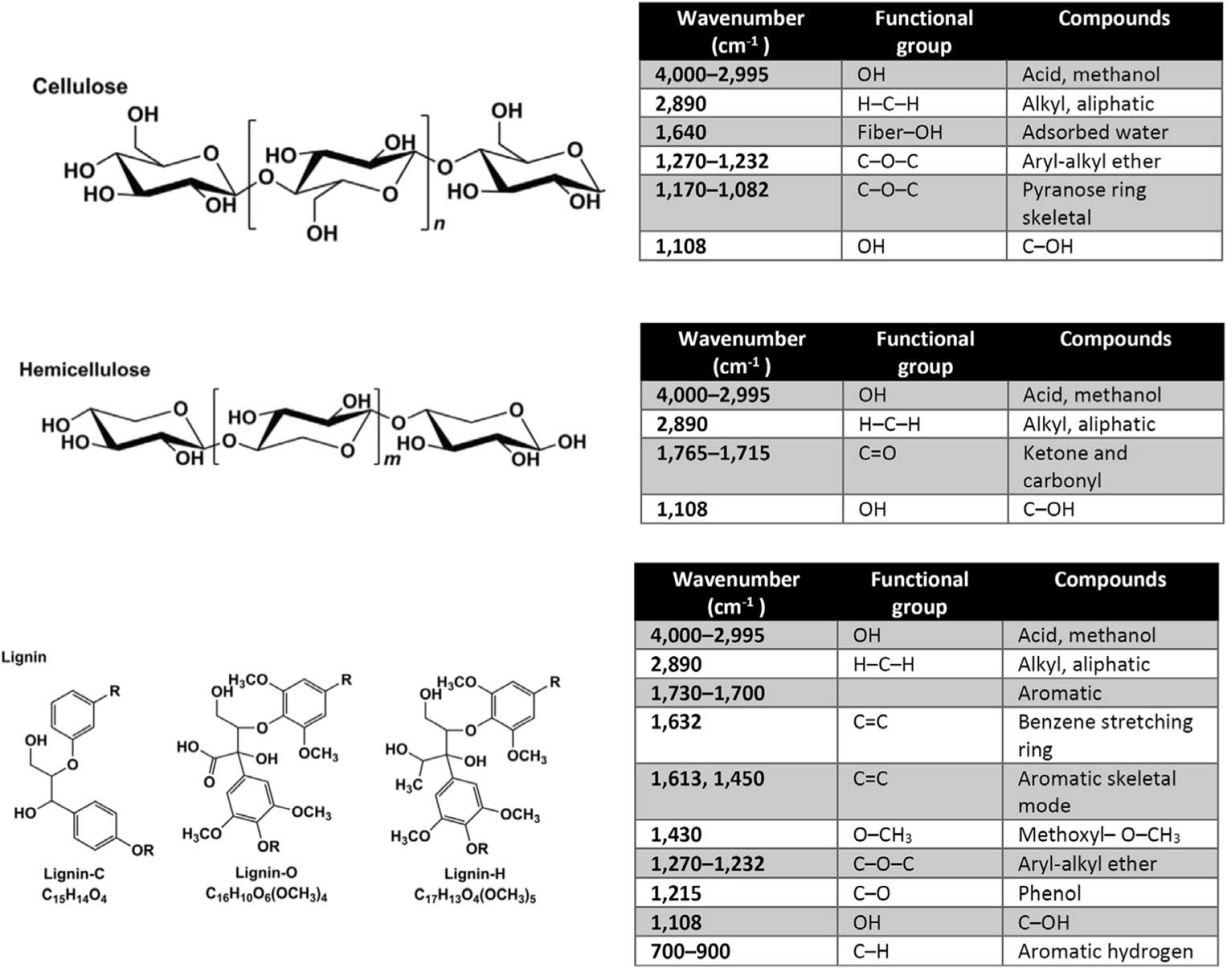
Absorption bands for functional groups of cellulose, hemicellulose and lignin [37, 38]

Figure 2b shows the FTIR spectra of black NOS before and after MB adsorption. It consists of several peaks such as O-H stretching at 3329 cm^−1^, C-H alkane stretching at 2923.63 cm^−1^, C=O carbonyl stretching at 1737.90 cm^−1^, and C=C aromatic groups at 1594.89 cm^−1^. After MB adsorption, the following peaks were shifted to higher wavenumber: -OH at 3339.71 cm^−1^, C=O stretching at 1740 cm^−1^, C=C at 1597.02 cm^−1^. Figure 3 shows the main functional groups and the corresponding bands for cellulose, hemicellulose and lignin [37, 38].

#### Bulk, particle density, porosity and pH_solution_

Bulk density of an adsorbent is the ratio of mass of the material divided by the volume occupied [39]. The bulk density of the green NOS was recorded to be 0.968 g/mL while for the black NOS was 0.967 g/mL; indicating high organic matter in the adsorbent [38]. The particle density of green NOS (1.428 g/mL) was higher than black NOS (1.25 g/mL). Porosity is another parameter that gives the amount of total pore spaces or void surface area. The total pore spaces percentages were found to be 32.21% for the green NOS and 22.64% for the black NOS. These values suggest how much of MB dye can pass through the adsorbents. The pH_solution_ showed the acidic nature for both black and green NOS, which were 5.29 and 5.34, respectively.

### Effects of different adsorption parameters

#### Effect of pH

**Fig. 4:**
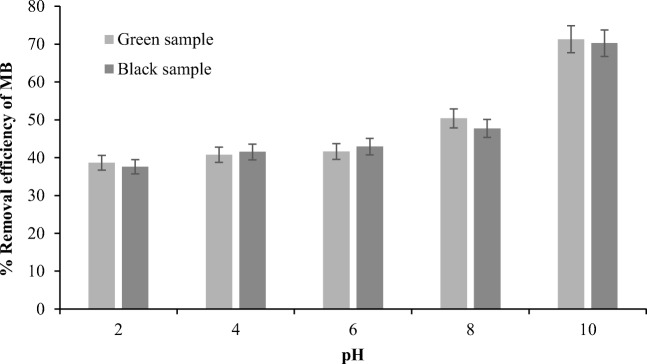
Effect of pH on the MB adsorption by green and black NOS at 25 °C

It has been suggested that pH affects the dye removal performance due to the difference of ionic charges [40]. It was evident that the adsorption of MB is pH dependent. Figure 4 shows that the MB adsorption increased from around 38% to approximately 70% for both black and green NOS when the pH increased from 2 to 10. This indicates that the adsorption process preferred basic environment. This might be due to the dominant negative charge on the surface deprotonation that results from the presence on hydroxyl ions on the adsorbent’s surface [41]. Manna et al. [42] stated that the low removal efficiency could be explained by the repulsion between the protonation of hydroxyl and/or carbonyl groups on the adsorbent surface and the MB positive ions. In other words, the electrostatic attractive force between the adsorbent surface and cation dye was very low due to the existence of hydrogen ions that compete on the active sites with the adsorbent. According to Mulugeta and Lelisa [43], the negative charges on the adsorbent surface facilitate and provide more active sites for the adsorption. This favorable adsorption at pH 10 was also reported and suggested by different studies [44–46].

#### Effect of initial MB concentration

**Fig. 5:**
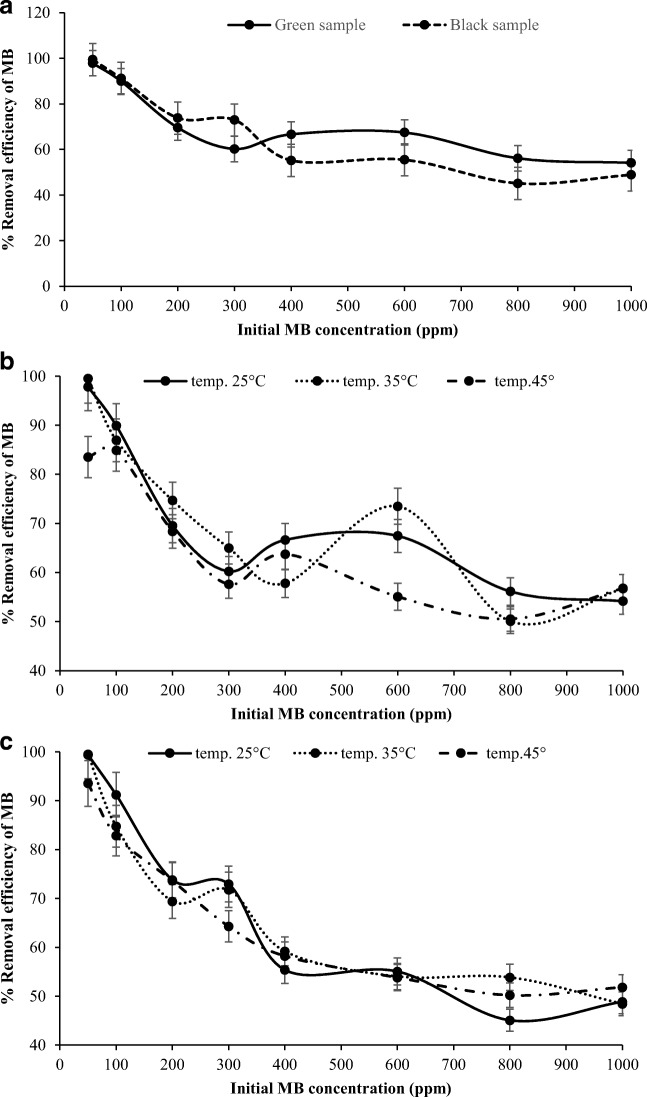
**a** Effect of concentration on green and black NOS on the removal of MB at different concentrations at 25 °C, (**b**) Effect of temperature on green NOS, and (**c**) black NOS at different temperatures 25, 35, and 45 °C

Figure 5a shows the effect of initial concentration on MB adsorption onto green and black NOS. The percentages of MB removal were observed to be the highest at 50 ppm with 97.83% and 99.39% for green and black NOS, respectively. There was a slight decrease of MB uptake, which reached to 89.93% and 91.22% for green and black NOS, respectively. The percentage of dye removal decreased accordingly with higher concentrations. This is due to the availability of the vacant binding sites on the adsorbent surface [47]. The decreasing effect of MB removal was continued to reach 69.55% and 73.81% at 200 ppm for green and black NOS, respectively. At 300 ppm, the decreasing effect continued for green NOS to 60.23%, while it remained constant for the black NOS. After this concentration, the behavior of MB removal for both adsorbents was varied. The removal efficiency of green NOS was raised again to 66.64% at 400 ppm and to 67.46% at 600 ppm as depicted in Fig. 5a, and then it decreased again to 56.13% at 800 ppm and to 54.16% at 1000 ppm. For the black NOS, Fig. 5a also shows that the removal efficiency was decreased to 55.18% at 400 ppm, and it continued to decrease until it reached 45.08% at 800 ppm, and then slight increase in the MB removal was observed at 1000 ppm, which reached to 48.88%. This gradual decrease of the MB removal and low de-colorization percentage results at high concentration may be ascribed to the inhibitory effects of high dye concentration [48], and to the increase of driving force for mass transfer, as well as to the lack of required active sites for the adsorption process [49]. Janoš et al. [50] suggested the low concentrations of the MB dye can enhance and improve the adsorption process, whereas, high initial concentrations solubilized the dyes and kept them in solution. Moreover, higher percentage of MB adsorption and faster attainment of adsorption equilibrium was indicated at lower initial dye concentration. This finding could be attributed to the spontaneous accessibility of the unoccupied active sites on the surface of the adsorbent by the MB molecules in the lower initial concentration [51]. This study on the removal of MB at different concentrations confirms the results of the previous studies, which shows the less-availability of the active sites on the surface of the adsorbent at high initial concentrations of the dye. Similar decrease of MB removal was observed by Al-Ghouti et al. [52].

#### Effect of solution temperature

Figure 5b, c shows the effect of solution temperatures on MB adsorption onto black and green NOS at different initial MB concentrations. Overall, it was observed that the amount of MB adsorbed decreased at higher temperature. The maximum MB adsorbed was recorded at 35 °C, followed by 25 °C. The black NOS showed similar behavior as that of green NOS. At 25 °C, the removal uptake percentage was decreased linearly from 99.39% to 45.08% with increasing the concentration from 50 to 800 ppm, while it increased to 48.88% at higher concentration. At 35 °C, the removal efficiency decreased constantly from 99.49 to 48.43% with increasing the concentration, and similar trend was noticed at 45 °C in which it decreased from 93.55 to 51.84%. However, at 25 °C, the highest adsorption of MB was recorded in low concentrations of black and green NOS at 50 and 100 ppm, respectively. This could be due to the increase in the solubility of the dye at higher temperature that perhaps decreased the interactions between the adsorbate and the adsorbent [53]. Furthermore, it can also be due to the increase in the intrinsic viscosity of the solution [54]. In general, the observations indicate that the removal efficiency of the black and green NOS decreased with increase in temperature due to the increase in the mobility of the MB ions, which will escape from the surface of the adsorbent [55]. Similar results were observed by Manna et al. [42].

### Adsorption isotherm

The adsorption parameters of the three adsorption isotherm models are summarized in Table 3. The best-fit model was selected based on correlation coefficient (R^2^) value, RMSE, SSE, and Chi-squares (*x*^*2*^) [56]. Freundlich and Langmuir models were among the best fit for the equilibrium data. For the black NOS, the q_m_ suggested that the adsorption process is more preferable at a high temperature, While for the green NOS, q_m_ values were observed to be highest at either 25 °C or 45 °C (625 mg/g), which perhaps indicates the process either prefers a low or high temperature. On the other hand, when comparing R^2^ value of Freundlich isotherm for both black and green NOS, it is clear that the values were closer to one; reaching up to 0.992 and 0.995, respectively. According to Tang et al. [8], when the value of 1/n is between 0.1 and 0.5 (0.1 < 1/*n* ≤ 0.5); the adsorption process is assumed to be easy. In the current study, the values of 1/n were between 0.312–0.405 for the green NOS samples, and 0.286–0.451 for the black NOS, which suggests adsorption process was easy onto the surface of NOS, which is due to the distribution of energy sites. Furthermore, it is also evident that the adsorption process was more efficient using black NOS in contrast with the green NOS. In addition, the values of K_f_ for both green and black NOS decreased with increasing the temperature. The maximum adsorption capacity for black NOS was recorded at 25 °C with 64.2 mg/g, while for green NOS it was found to be at 35 °C with 58.3 mg/g. Thus, it is apparent that the adsorption process was well described by Freundlich model, which describes that the process took place on a heterogeneous surface by a uniform energy distribution and reversible adsorption [34, 57]. This could be due to the presence of various functional groups as well as the elemental composition of NOS that perhaps might have facilitated the adsorption process. Previous researchers showed that the maximum adsorption capacity of MB was 111.1 mg/g using cucumber peels, 309.6 mg/g using shaddock peel [58], and 109.9 mg/g using banana leaves [8, 59]. Another dimensionless parameter that can be calculated is separation factor (R_L_), which can detect the favorability of the adsorption process. According to Pang et al. [59], R_L_ values can be calculated using the following equation:

**Table 3:** Langmuir Freundlich and Dubinin-Radushkevish Parameters for Adsorption of MB on black and green NOS

Adsorbent	Isotherm	Parameter	Temperature, ^o^C		
			25	35	45
Black NOS	Langmuir	q_m_ (mg/g)	476	556	556
		K_L_ (L/mg)	0.0116	0.00830	0.00680
		RMSE	1.39	1.46	1.55
		SSE	2.14	2.24	2.20
		X^2^	0.143	0.235	0.200
		R^2^	0.877	0.873	0.814
	Freundlich	1/n	0.286	0.301	0.451
		K_f_ ((mg/g)(L/g)^n^)	64.2	56.5	26.0
		RMSE	0.0321	0.0344	0.0389
		SSE	0.0133	0.0156	0.0177
		X^2^	0.771	0.889	0.879
		R^2^	0.956	0.883	0.992
	Dubinin-Radushkevish	B_D_ (mol^2^/kJ^2^)	0.00042	0.00066	0.00045
		E (kJ/mol)	34.50	27.52	33.33
		RMSE	0.0411	0.0444	0.0377
		SSE	0.0155	0.0186	0.0201
		X^2^	0.781	0.899	0.889
		R^2^	0.8900	0.9100	0.8688
Green NOS	Langmuir	q_m_ (mg/g)	625	556	625
		K_L_ (L/mg)	0.00725	0.00928	0.00521
		RMSE	2.900	2.900	2.790
		SSE	0.1780	0.1900	0.1800
		X^2^	0.9870	0.8790	0.9930
		R^2^	0.7520	0.7620	0.8450
	Freundlich	1/n	0.4050	0.3120	0.5850
		K_f_ ((mg/g)(L/g)^n^)	39.50	58.30	13.00
		RMSE	0.0401	0.0398	0.0422
		SSE	0.0142	0.0122	0.0133
		X^2^	0.6790	0.6890	0.6650
		R^2^	0.9530	0.8230	0.9950
	Dubinin-Radushkevish	B_D_ (mol^2^/kJ^2^)	0.0024	0.0017	0.0025
		E (kJ/mol)	14.43	17.15	14.14
		RMSE	0.0321	2.920	0.0389
		SSE	0.0133	0.2030	0.0177
		X^2^	0.7710	0.8890	0.8790
		R^2^	0.9591	0.6300	0.9256

8 $$ {\mathrm{R}}_{\mathrm{L}}=\left(1/1+{\mathrm{K}}_{\mathrm{L}}.{\mathrm{C}}_0\right) $$

Where K_L_ is Langmuir constant and C_o_ is the initial concentration of MB. When the value of R_L_ is between 0 and 1, the adsorption process is favorable on the adsorbent, greater than 1 implies the process is unfavorable [60]. The calculated values of R_L_ for both black and green NOS were between 0 and 1, further indicating the favorability of the adsorbent to remove the MB from water. Table 4 compares the MB adsorption capacity among other adsorbents from previous studies.

**Table 4:** Comparison of MB adsorption capacity among other adsorbents from the previous studies and the present study

Adsorbent	Adsorption capacity (mg/g)	Reference
Black NOS	476	Current study
Green NOS	625	Current study
Porous chitin sorbent	92.6	[61]
Activated carbon from banana trunk	167	[62]
Activated biochar from barley malt bagasse	161	[63]
Algerian Kaolin	52.8	[64]
Acid washed black cumin seed	74	[65]
Bamboo hydrochar	656	[66]
Cotton based flexible carbon fiber aerogel	102	[67]
mesoporous birnessite-type manganese oxide	113	[59]
Activated Carbon from *Ficus carica* bast	47.6	[49]

B_D_ in the Dubinin–Radushkevich isotherm represents the mean free energy (E) of adsorption per mole of the adsorbate (kJ/mol), and could be a useful magnitude for interpretation of the adsorption mechanisms of MB onto NOS, which can be obtained from the following equation:

9 $$ E=\frac{1}{\sqrt{2{B}_D}} $$

If E is less than 8 kJ/mol, then the adsorption process may be affected by physical attraction. In the case of E is between 8 and 16 kJ/mol, the adsorption process is governed by ion exchange mechanism, and for the value of E greater than16 kJ/mol may be dominated by particle diffusion phenomenon.

In order to find the most-stable MB adsorbed onto the surfaces of the black NOS and green NOS and to distinguish between the physical and chemical adsorption of MB ions, E_D_ (per molecule of adsorbate) from the Dubinin-Radushkevich adsorption model was used. For example, in the MB adsorbed onto NOS system, the E_D_ was calculated according to,

10 $$ \Delta  {E}_D=\left[{E}_{MB\  adsorbed\  on\  NOS}-\left({E}_{NOS}+{E}_{MB}\right)\right] $$

**Fig. 6:**
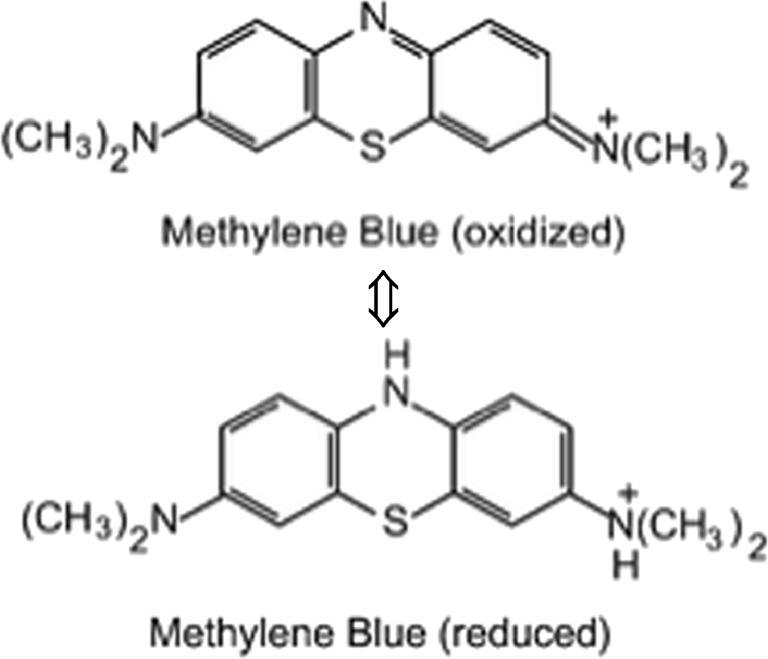
MB equilibrium of reduction and oxidation

Where E_MB adsorbed on NOS_, E_NOS_ and E_MB_ are total energies of the MB ions adsorbed NOS system, the NOS surface and single MB ions, respectively. Accordingly, it can be suggested that the MB ions are easy to form onto the surface of the green NOS with the lowest free energy (E) of adsorption per mole of the adsorbate (14.43 kJ mol^−1^ at 25 °C); suggesting that the adsorption process is governed by ion exchange mechanism. This variation on E values could be related to the adsorption profile of MB ions onto the surface of the adsorbents and the MB oxidation and reduction. Once the MB adsorbed on the surface of the adsorbent may undergo an equilibrium of reduction and oxidation as shown in Fig. 6. Accordingly, the configuration would be changed according to the state of MB and the amount of energy required for adsorption.

### Adsorption thermodynamics

Table 5 shows the thermodynamics parameter of MB adsorption onto green and black NOS. It was shown that the values of Gibbs free energy (ΔG°) was decreased as temperature increased for both adsorbents, indicating the spontaneous nature of the adsorption process and the favorability of the process [8, 68]. The value of ΔS° was 82.35 J/mol.K; signifying the increase in the randomness of the adsorption system [15]. The negative value of ΔH° for both adsorbents suggests that the adsorption process followed an exothermic pathway.

**Table 5:** Thermodynamics parameter of MB adsorption by green and black NOS

Temperature (K)	ΔG^o^ (kJ/mol)	ΔH^o^ (kJ/mol)	ΔS^o^ (J/mol.K)
Green NOS			
298	−12.67	−12.67	82.35
308	−38.05		
318	−38.87		
Black BOS			
298	−21.45	−21.28	108.6
308	−21.45		
318	−21.46		

### Adsorption mechanisms

Investigating the adsorption mechanisms and adsorption-driving forces of organic molecules from aqueous solution to adsorbent’s surface are of great importance. Such adsorption-driving forces might include the followings: (i) MB hydrophobicity that depresses MB from remaining in the bulk aqueous solution and (ii) specific MB-NOS surface attractions arising hydrogen bonding, electrostatic attraction, and/or electron donor-acceptor interactions [69]. Such adsorption-driving forces are also described as the formation of inner sphere complexes since the NOS surface and MB approach one another close enough to facilitate overlap of the orbitals responsible for bonding. All these adsorption-driving forces are forms of physisorption since the MB-NOS surface associations do not involve the formation of new bonds.

Accordingly, the MB structure, the composition of the NOS, and the experimental conditions of the MB solution exchanging with NOS were considered. NOS is lignocellulosic biomass, which has cellulose with varying content between 30 to 34%, hemicellulose content from 21 to 28%, and lignin from 21 to 25% as major components. Based on the FTIR spectra of the black and green NOS (Fig. 2), the major functional groups on the NOS surface are hydroxyl, ether, and carbonyl.

**Fig. 7:**
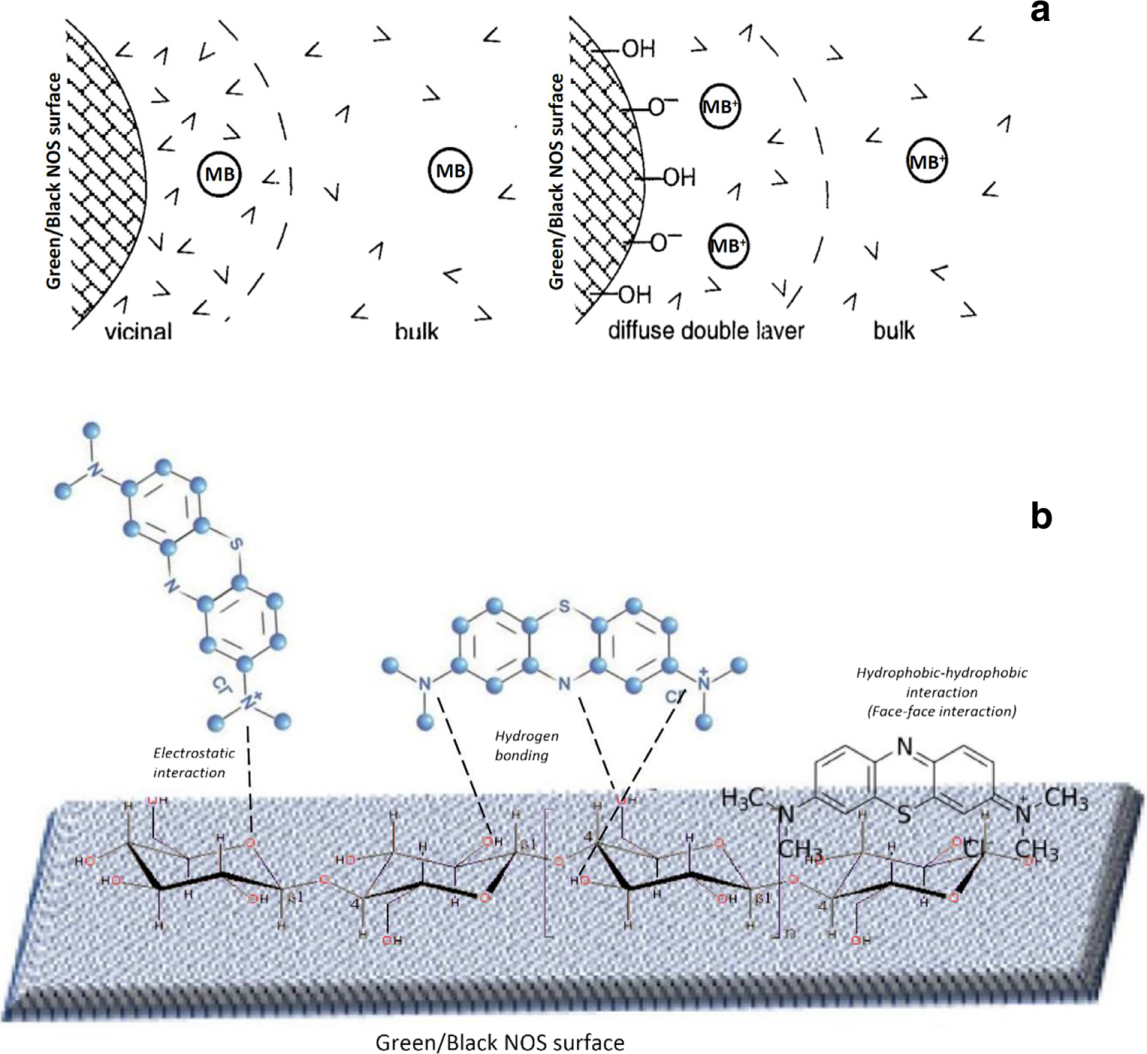
**a** schematic views of various ways in which MB adsorbed onto NOS solids: MB partitioning from water to the layer of “vicinal water” adjacent to NOS surface (left), and adsorption of MB^+^ molecules from water to NOS charged surfaces due to electrostatic attractions (right) [69], and **b** possible MB interactions onto the green/black NOS surface

Here, MB is attracted to NOS by Van der Waals (vdW) forces, stronger attractions per unit surface area of NOS are also observed as corresponding functional groups including hydroxyl, ether, and carbonyl are included on the NOS surface and MB that are capable of forming H-bond. However, the surface attraction energy for an H-donor and H-acceptor adsorbate like water are very strong; inferring that NOS might prefer to bind water over MB molecules. Fig. 7 shows the schematic views of various ways in which MB adsorbed onto NOS solids: MB partitioning from water to the layer of “vicinal water” adjacent to NOS surface, and adsorption of MB^+^ molecules from water to NOS charged surfaces due to electrostatic attractions, and the possible MB interactions onto the green/black NOS surface.

### Statistical analysis

Statistically designed experiments were used to examine the overall effect of the parameters. In order to check the significance of each of the coefficients and to understand the pattern of the mutual interactions between the tested parameters, the *P* values were used. The smaller the magnitude of P values and the larger the magnitude of F-test value, the higher the significance of the corresponding coefficient. As shown in Table 6, that the *p* values of both adsorbents for pH parameter is smaller than 0.05, in which the p value of black NOS is 2.078 × 10^−5^ and the p value of green NOS is 2.975 × 10^−5^, indicating that the samples are significantly different. The p values for the effect of pH for black and green NOS were 2.078 × 10^−5^ and 2.975 × 10^−5^, respectively, which are below the *P* = 0.05; showing significance exists as shown in Table 6. In addition, the P value for concentration were smaller than 0.05 implying the significant difference exists. All F-values for all parameter were high.

**Table 6:** ANOVA analysis for MB adsorption onto black NOS and green NOS

Parameter	F-value	*P*-value	F-crit
pH (black NOS)	78.51	2.078 × 10^−5^	5.318
Initial MB concentration (black NOS)	6.731	2.63 × 10^−5^	2.130
pH (green NOS)	71.17	2.975 × 10^−5^	5.318
Initial MB concentration (green NOS)	89.23	1.27 × 10^−15^	2.423

## Conclusion

The result obtained successfully demonstrates the potential of using black and green NOS as suitable adsorbents for the removal of MB from water. The present study was conducted to test the efficiency of the NOS as a low-cost and economic adsorbent for the adsorption and removal of MB dye from water. It was found that alkaline conditions increased the percentage removal of the MB dye as the highest efficiency of removal was observed at pH 10. The adsorption process was best fitted by Freundlich model, which explain the multilayer sorption on heterogeneous surfaces. Furthermore, the negative values of ΔG° indicated the spontaneous nature while the positive value of ΔS° explained the increase in the randomness. From negative value of ΔH°, it seemed the process followed an exothermic pathway. The Freundlich adsorption capacity was found to be 64.16 (mg/g)(L/mg)^1/n^ at 25 °C using black NOS. It was found that the nano-sized material can enhance the adsorption capabilities compared with other adsorbents, and it exhibits a potential to be used as a commercial adsorbent to treat water efficiently and cost-effectively.

## References

[1] Mansoorian HJ, Mahvi AH, Alizade M. Equilibrium and synthetic studies of methylene blue dye removal using ash of walnut shell. J Health Field. 2013;1(3):38–45.

[2] Ashrafi SD, Rezaei S, Forootanfar H, Mahvi AH, Faramarzi MA (2013). The enzymatic decolorization and detoxification of synthetic dyes by the laccase from a soil-isolated ascomycete, Paraconiothyrium variabile. Int Biodeter Biodegr.

[3] Lu X, Liu L, Liu R, Chen J (2010). Textile wastewater reuse as an alternative water source for dyeing and finishing processes: A case study. Desalination.

[4] Zheng H, Zhang J, Yan J, Zheng L (2016). An industrial scale multiple supercritical carbon dioxide apparatus and its eco-friendly dyeing production. J CO2 Util.

[5] Pang YL, Abdullah AZ (2013). Current status of textile industry wastewater management and research progress in Malaysia: A review. Clean Soil Air Water.

[6] Raman CD, Kanmani S (2016). Textile dye degradation using nano zero valent iron: A review. J Environ Manag.

[7] Ehrampoush MH, Moussavi GHR, Ghaneian MT, Rahimi S, Ahmadian M (2010). Removal of methylene blue (MB) dye from textile synthetic wastewater using TiO_2_/UV-C photocatalytic process. Aust J Basic Appl Sci.

[8] Tang R, Dai C, Li C, Liu W, Gao S, Wang C. Removal of methylene blue from aqueous solution using agricultural residue walnut Shell: equilibrium, kinetic, and thermodynamic studies. J Chem. 2017;2017:10.

[9] Peláez-Cid AA, Herrera-González AM, Salazar-Villanueva M, Bautista-Hernández A (2016). Elimination of textile dyes using activated carbons prepared from vegetable residues and their characterization. J Environ Manag.

[10] Singh K, Arora S (2011). Removal of synthetic textile dyes from wastewaters: a critical review on present treatment technologies. Crit Rev Environ Sci Technol.

[11] Hussein A, Scholz M (2017). Dye wastewater treatment by vertical-flow constructed wetlands. Ecol Eng.

[12] Qu X, Brame J, Li Q, Alvarez PJ (2012). Nanotechnology for a safe and sustainable water supply: enabling integrated water treatment and reuse. Acc Chem Res.

[13] Šostar-Turk S, Petrinić I, Simonič M (2005). Laundry wastewater treatment using coagulation and membrane filtration. Resour Conserv Recycl.

[14] Wang Z, Xue M, Huang K, Liu Z. Textile dyeing wastewater treatment. Advances in Treating Textile Effluent, Prof. Peter Hauser (Ed.), InTech. 2011. Available from http://www.intechopen.com/books/advances-in-treating-textile-effluent/textile-dyeing-wastewatertreatment.

[15] Shakoor S, Nasar A (2017). Adsorptive treatment of hazardous methylene blue dye from artificially contaminated water using cucumis sativus peel waste as a low-cost adsorbent. Groundw Sustain Dev.

[16] Altaher H, ElQada E (2011). Investigation of the treatment of colored water using efficient locally available adsorbent. Int J Energy Environ.

[17] Yakout SM, El-Deen GS (2016). Characterization of activated carbon prepared by phosphoric acid activation of olive stones. Arab J Chem.

[18] Spahis N, Addoun A, Mahmoudi H, Ghaffour N (2008). Purification of water by activated carbon prepared from olive stones. Desalination.

[19] Matos M, Barreiro MF, Gandini A (2010). Olive stone as a renewable source of biopolyols. Ind Crop Prod.

[20] Albroomi HI, Elsayed MA, Baraka A, Abdelmaged MA (2017). Batch and fixed-bed adsorption of tartrazine azo-dye onto activated carbon prepared from apricot stones. Appl Water Sci.

[21] Rizzi V, D'Agostino F, Fini P, Semeraro P, Cosma P (2017). An interesting environmental friendly cleanup: the excellent potential of olive pomace for disperse blue adsorption/desorption from wastewater. J Dyes Pigm.

[22] Martín-Lara MA, Blázquez G, Trujillo MC, Pérez A, Calero M (2014). New treatment of real electroplating wastewater containing heavy metal ions by adsorption onto olive stone. J Clean Prod.

[23] Stagnaro SM, Volzone C, Huck L (2015). Nanoclay as adsorbent: evaluation for removing dyes used in the textile industry. Procedia Mater Sci.

[24] Anjum M. Miandad R. Waqas M. Gehany F. Barakat MA. Remediation of wastewater using various nano-materials. Arab J Chem. 2019;12:4897–919.

[25] Saravanan C, Rajesh R, Kaviarasan T, Muthukumar K, Kavitake D, Shetty PH (2017). Synthesis of silver nanoparticles using bacterial exopolysaccharide and its application for degradation of azo-dyes. Biotechnol Rep.

[26] Girgis BS, Attia AA, Fathy NA (2011). Potential of nano-carbon xerogels in the remediation of dye-contaminated water discharges. Desalination.

[27] Kyzas GZ, Matis KA (2015). Nanoadsorbents for pollutants removal: a review. J Mol Liq.

[28] Alimohammadi M, Saeedi Z, Akbarpour B, Rasoulzadeh H, Yetilmezsoy K, Al-Ghouti MA, Khraisheh M, McKay G (2017). Adsorptive removal of arsenic and mercury from aqueous solutions by Eucalyptus leaves. Water Air Soil Pollut.

[29] Bohli T, Fiol N, Villaescusa I, Ouederni A. Adsorption on activated carbon from olive stones: kinetics and equilibrium of phenol removal from aqueous solution. J Chem Eng Process Technol. 2013;04(06):165–9.

[30] Tan KB, Vakili M, Horri BA, Poh PE, Abdullah AZ, Salamatinia B (2015). Adsorption of dyes by nanomaterials: recent developments adsorption mechanisms. Sep Purif Technol.

[31] Galanakis CM (2011). Olive fruit dietary fiber: components, recovery, and applications. Trends Food Sci Technol.

[32] Aziz A, Ouali MS, Elandaloussi EH, De Menorval LC. Lindheimer M. (2009). Chemically modified olive stone: A low-cost sorbent for heavy metals and basic dyes removal from aqueous solutions. J Hazard Mater.

[33] Ramesh ST, Gandhimathi R, Badabhagni N, Nidheesh PV. Removal of Cd (II) from aqueous solution by adsorption onto coir pith, an agricultural solid waste: batch experimental study. Environ Eng Manag J. 2011;10(11):1667–73.

[34] Molavi H, Hakimian A, Shojaei A, Raeiszadeh M (2018). Selective dye adsorption by highly water stable metal-organic framework: long term stability analysis in aqueous media. Appl Surf Sci.

[35] Lessa EF, Gularte MS, Garcia ES, Fajardo AR (2017). Orange waste: A valuable carbohydrate source for the development of beads with enhanced adsorption properties for cationic dyes. Carbohydr Polym.

[36] Marković S, Stanković A, Lopičić Z, Lazarević S, Stojanović M, Uskoković D (2015). Application of raw peach shell particles for removal of methylene blue. J Environ Chem Eng.

[37] Morán JI, Alvarez VA, Cyras VP, Vázquez A (2008). Extraction of cellulose and preparation of nanocellulose from sisal fibers. Cellulose.

[38] Zhou X, Broadbelt LJ, Vinu R (2016). Chapter two - mechanistic understanding of thermochemical conversion of polymers and Lignocellulosic biomass. Adv Chem Eng.

[39] Abdullah E, Geldart D (1999). The use of bulk density measurements as flowability indicators. Powder Technol.

[40] Boumchita S, Lahrichi A, Benjelloun Y, Lairini S, Nenov V, Zerrouq F (2017). Application of Peanut shell as a low-cost adsorbent for the removal of anionic dye from aqueous solutions. J Mater Environ Sci.

[41] Malekbala MR, Hosseini S, Yazdi SK, Soltani SM, Malekbala MR (2012). The study of the potential capability of sugar beet pulp on the removal efficiency of two cationic dyes. Chem Eng Res Des.

[42] Manna S, Roy D, Saha P, Gopakumar D, Thomas S (2017). Rapid methylene blue adsorption using modified lignocellulosic materials. Process Saf Environ Prot.

[43] Mulugeta M, Lelisa B. Removal of methylene blue (Mb) dye from aqueous solution by bioadsorption onto untreated Parthenium hystrophorous weed. Modern Chem App. 2014;2:146.

[44] Ovando-Medina VM, Díaz-Flores PE, Martínez-Gutiérrez H, Moreno-Ruiz LA, Antonio-Carmona ID, Hernández-Ordoñez M (2014). Composite of cellulosic agricultural waste coated with semiconducting polypyrrole as potential dye remover. Polym Compos.

[45] Zhang W, Yang H, Dong L, Yan H, Li H, Jiang Z, Cheng R (2012). Efficient removal of both cationic and anionic dyes from aqueous solutions using a novel amphoteric straw-based adsorbent. Carbohydr Polym.

[46] Hamza W, Dammak N, Hadjltaief H, Eloussaief M, Benzina M (2018). Sono-assisted adsorption of Cristal violet dye onto Tunisian Smectite clay: characterization, kinetics and adsorption isotherms. Ecotoxicol Environ Saf.

[47] Vijayaraghavan K, Yun YS (2008). Biosorption of CI reactive black 5 from aqueous solution using acid-treated biomass of brown seaweed Laminaria sp. Dyes Pigments.

[48] Anjaneya O, Souche SY, Santoshkumar M, Karegoudar TB (2011). Decolorization of sulfonated azo dye Metanil yellow by newly isolated bacterial strains: Bacillus sp. strain AK1 and Lysinibacillus sp. strain AK2. J Hazard Mater.

[49] Pathania D, Sharma S, Singh P (2017). Removal of methylene blue by adsorption onto activated carbon developed from Ficus carica bast. Arab J Chem.

[50] Janoš P, Buchtova H, Rýznarová M (2003). Sorption of dyes from aqueous solutions onto fly ash. Water Res.

[51] Marrakchi F, Auta M, Khanday WA, Hameed BH (2017). High-surface-area and nitrogen-rich mesoporous carbon material from fishery waste for effective adsorption of methylene blue. Powder Technol.

[52] Al-Ghouti M, Khraisheh M, Allen S, Ahmad M (2003). The removal of dyes from textile wastewater: a study of the physical characteristics and adsorption mechanisms of diatomaceous earth. J Environ Manag.

[53] Obaid MH (2011). Thermodynamic study of adsorption cationic methylene blue dye on the plant residue. Kufa J Chem.

[54] Pekel N, Güven O (2002). Solvent, temperature and concentration effects on the adsorption of poly (n-butyl methacrylate) on alumina from solutions. Turk J Chem.

[55] Abdel A-KM, Francis RM, A. (2017). Exploring the adsorption behavior of cationic and anionic dyes on industrial waste shells of egg. J Environ Chem Eng.

[56] Ooi J, Lee LY, Hiew BYZ, Thangalazhy-Gopakumar S, Lim SS, Gan S (2017). Assessment of fish scales waste as a low cost and eco-friendly adsorbent for removal of an azo dye: equilibrium, kinetic and thermodynamic studies. Bioresour Technol.

[57] Hazzaa R, Hussein M (2015). Cationic dye removal by sugarcane bagasse activated carbon from aqueous solution. Glob Nest J.

[58] Liang J, Wu J, Li P, Wang X, Yang B (2012). Shaddock peel as a novel low-cost adsorbent for removal of methylene blue from dye wastewater. Desalin Water Treat.

[59] Pang J, Fu F, Ding Z, Lu J, Li N, Tang B (2017). Adsorption behaviors of methylene blue from aqueous solution on mesoporous birnessite. J Taiwan Inst Chem Eng.

[60] He J, Cui A, Deng S, Chen JP (2018). Treatment of methylene blue containing wastewater by a cost-effective micro-scale biochar/polysulfone mixed matrix hollow fiber membrane: performance and mechanism studies. J Colloid Interface Sci.

[61] Cao Y, Pan Z, Shi Q, Yu J (2018). Modification of chitin with high adsorption capacity for methylene blue removal. Int J Biol Macromol.

[62] Danish M, Ahmad T, Majeed S, Ahmad M, Ziyang L, Pin Z, Iqubal SS (2018). Use of banana trunk waste as activated carbon in scavenging methylene blue dye: kinetic, thermodynamic, and isotherm studies. Bioresour Technol Rep.

[63] Franciski M, Peres E, Godinho M, Perondi D, Foletto E, Collazzo G, Dotto G (2018). Development of CO2 activated biochar from solid wastes of a beer industry and its application for methylene blue adsorption. Waste Manag.

[64] Mouni L, Belkhiri L, Bollinger J, Bouzaza A, Assadi A, Tirri A, Remini H (2018). Removal of methylene blue from aqueous solutions by adsorption on kaolin: kinetic and equilibrium studies. Appl Clay Sci.

[65] Siddiqui SI, Rathi G, Chaudhry SA (2018). Acid washed black cumin seed powder preparation for adsorption of methylene blue dye from aqueous solution: thermodynamic, kinetic and isotherm studies. J Mol Liq.

[66] Qian W, Luo X, Wang X, Guo M, Li B (2018). Removal of methylene blue from aqueous solution by modified bamboo hydrochar. Ecotoxicol Environ Saf.

[67] Li Z, Jia Z, Ni T, Li S (2017). Adsorption of methylene blue on natural cotton based flexible carbon fiber aerogels activated by novel air-limited carbonization method. J Mol Liq.

[68] Ashrafi SD, Kamani H, Jaafari J, Mahvi AH (2016). Experimental design and response surface modeling for optimization of fluoroquinolone removal from aqueous solution by NaOH-modified rice husk. Desalin Water Treat.

[69] Schwarzenbach RP, Gschwend PM, Imboden DM (2003). Environmental organic chemistry.

